# Adaptation and evaluation of the program based on Specialized Coordinated Care for First Psychotic Episode in Chile - OnTrackChile: preliminary results of implementation fidelity

**DOI:** 10.1192/j.eurpsy.2025.497

**Published:** 2025-08-26

**Authors:** R. Alvarado, M. S. Burrone, F. Leyton, M. J. Jorquera, S. Schilling, M. Gómez, I. Bello, J. Ramírez, D. Arancibia, K. Fader, S. Conover, L. H. Yang, E. Susser, L. Dixon, L. J. Cabassa

**Affiliations:** 1 School of Medicine, Faculty of Medicine, Universidad de Valparaíso, Valparaiso; 2 Instituto de Ciencias de la Salud, Universidad de O´Higgins, Rancagua; 3 Faculty of Medicine, Universidad de Chile, Santiago, Chile; 4 New York State Psychiatric Institute, New York, United States; 5 Faculty of Health Sciences, Universidad Central, Santiago, Chile; 6 Mailman School of Public Health, Columbia University; 7 Silberman School of Social Work, Hunter College; 8 Department of Social and Behavioral Sciences, School of Global Public Health New York University; 9 Columbia University Vagelos College of Physicians and Surgeons, New York; 10 Brown School of Social Work at Washington University in St. Louis, St. Louis, United States

## Abstract

**Introduction:**

In high-income countries, Coordinated Specialized Care for First Psychotic Episode (FEP) programs have been shown to be effective in reducing symptoms and disability. Chile guarantees universal access to these services through a national policy, but previous research indicates that evidence-based approaches are not used. Our team adapted the OnTrackNY (OTNY) program to the Chilean context, called OnTrackChile (OTCH), to evaluate its effectiveness and implementation. This summary presents preliminary results on model fidelity, one of the primary outcomes of the study.

**Objectives:**

To evaluate the fidelity of the implementation of the OTCH program in comparison with usual care services for FEP, analyzing its compliance in 18 key domains of care and its alignment with the National Mental Health Plan.

**Methods:**

A fidelity scale was designed to guide data collection on OTCH implementation. The scale included the name and definition of each domain, along with a set of expectations. The scale assessed 18 key domains, such as staffing, team integration, communication, burden of care, service flexibility, crisis, treatment planning, prescribing, care management, working with families, and education and employment support, among others. The scale was applied in the 5 OTCH intervention sites and 8 control sites that maintained usual care included in the cluster-randomized controlled clinical trial.

**Results:**

OTCH sites met more than 80% of the criteria for the domains assessed in the fidelity scale, which is more than twice the compliance observed in the control sites. In domains related to the usual functioning of the centers coincident with those established in the National Mental Health Plan, OTCH intervention sites exceeded compliance standards to control sites (p<0.001). In situations where the frequency of problems was similar between both types of centers, such as suicidal risk and risky substance use, OTCH centers showed significantly better performance (p<0.001) compared to control centers.

**Conclusions:**

The implementation of the OTCH model has not only allowed the introduction of specific aspects of the program, but has also improved the overall performance of the centers in key areas defined by the National Mental Health Plan. This suggests that the implementation of OTCH in the Chilean context is not only feasible, but can also improve the quality of community mental health care.

OnTrack Chile is funded by the U.S. National Institute of Mental Health (R01MH115502).

**Disclosure of Interest:**

None Declared

